# Cases from the Surgical Clinic of Prof. Moses Gunn, of Rush Medical College

**Published:** 1870-10

**Authors:** H. F. Chesbrough


ARTICLE III.—*Cases from the Surgical Clinic of Professor Moses Gunn, of Rush Medical College.* Reported by H. F. CHESBROUGH, M.D.*

I. Saturday, Dec. 10, 1869. L. S., of B——, Wisconsin, was brought before the class, by his physician, on account of a severe pain seated in the internal malleolus of the left leg. This young man had been unable to walk without crutches for about two years, and had applied to his physician to have his leg amputated, on account of the long continued and excessive pain. His attendant was unwilling to take the responsibility of amputating a limb which seemed perfectly sound to a casual observer, and so brought him to Chicago, to the College Clinic. Dr. Gunn spoke of the history of the case, comprising more than two years of severe persistent, paroxysmal pain, the tenderness on pressure, and the enlargement of the bone at that point, and pronounced the diagnosis of abscess of the bone. He then laid bare the tibia to a sufficient extent, trephined through the malleolus, and in the cancellar tissue of the bone, found an abscess the size of a hickory nut. It was full of well formed pus, which was distinctly visible on the sponge. The smooth walls of the abscess were also felt at bottom of the cavity made by the operation.

The wound was filled with lint and afterward with a wax plug, and allowed to fill up gradually, with granulations from the bottom. A letter was received some six weeks after from his attending physician, stating that the wound had run its natural course, had readily healed, and left the patient with a perfect recovery.

II. An old lady, aged 69, came from Wisconsin to get advice for a cancerous tumor of the breast. It was a scirrhus of large size, and was extirpated. The patient was very timid and the pulse weak and frequent, so that the æther had to be given with great caution. She was able to go home in a few days. * * Last week a letter was received from the husband, stating that the tumor began to grow in the cicatrix before the wound was entirely

* One reason for reporting these cases is, that the students who have seen the operations may know the results, thus adding much, not only to the interest, but also to the value of the clinic.

healed, and was now suppurating freely and very painful, and the patient in feeble health. This case then, as is so often the experience with scirrhus, was attended with only moderate success.

III. H. F., of Illinois, aged 18, presented himself on account of necrosis of the femur. He had contracted the disease while skating. The dead bone was taken away by cutting down upon it and removing it with forceps and chisel. The wound was filled with lint, and on the second day after the operation the plug of lint was removed, and one of wax, made to fit the wound perfectly, was put in its place. By this means the finger could be put into the wound, and the surgeon could feel, day by day, the progress of the case. After all the spiculæ of bone had ulcerated off, and the surface of exposed bone at the deepest part of the wound presented the smooth, velvety feel of healthy granulations, the patient was dismissed, and instructed to pare off a thin shaving from the end of the wax tent at every daily dressing. He was able to go home within ten days, with the certainty of a favorable result from the operation.

IV. On Jan. 22, 1870, came M. D., of Chicago, a young boy who had suffered from necrosis of the femur more than four years. His thigh presented the scars of dried up sinuses, and two openings, from which pus was still continually oozing. A small amount only of dead bone was removed, and the wound was kept open with a wax tent till early in March. Then the last piece of dead bone which could be felt by the finger came away, and the plug was gradually curtailed of its dimensions. Since then the sinuses have gradually closed, and the leg is perfectly healed.

V. C. S., with necrosis of the tibia, presented himself for an operation. He was treated as the others, and was perfectly cured in two months.

VI. On the 12th of Feb., 1870, B. R., of Chicago, an ill-conditioned, pale boy, appeared at the clinic, with extensive necrosis of the left femur. At the operation several small pieces of dead bone, and one large piece, two and a half inches long, very jagged and irregular, were removed. The cut was made between the inner hamstring muscles and the vastus internus, at the lower and inner part of the thigh. Of course the fibres of the latter muscle were partly divided. On account of the femoral artery the incision could not be carried upward along the femur, as far as the necrosis extended. Therefore a piece of wax was shaped to correspond with the shape and size of an index finger, bent at a right angle at the second joint, and this plug was inserted into the wound after suppuration had been established by the introduction of lint. At this time a jagged piece of dead bone could be felt at the deepest part of the incision, which evidently extended up the shaft of the femur. In about three weeks this piece could be felt coming down gradually, and the wax tent was cut off occasionally to accommodate the descent of the bone, until on the 30th day of April, eleven weeks after the operation, it became loose enough to remove with the forceps, and, when brought to light, proved to be even larger than the first piece. After this the wound was allowed to granulate rapidly from the bottom, and at the present writing, the lad is looking much improved, and the leg is perfectly sound.

On the text of these four cases, Prof. Gunn made a few remarks. He said that early in his practice he would operate for necrosis and employ the recognized after-treatment of filling the incision with lint and keeping the wound open as long as possible, to allow all the dead bone present to exfoliate and pass off. Yet with all the precautions possible it happened to him several times to have to cut a second time down on the same diseased bone. This led him to search for some remedy to obviate the disagreeable necessity of operating twice on the same case, and the wax plug was hit upon. Thus with its use in this case (VI,) the wound was kept open eleven weeks, until the second large fragment was pulled out, and then, by being gradually cut away at its distal end, it permits the wound to fill up from the bottom by granulations, and the gradual healing leaves a solid and perfect scar. The Professor added that after adopting this after-treatment in necrosis, it had never occurred to him to be forced to repeat the operation in the same case. In case III, the bottom of the wound was plainly felt by the finger, within ten days from the operation, to be covered by soft, smooth granulations, and then it was known that it was perfectly safe to let the patient go home. In case IV, the wound was kept open six weeks, while a few sharp points and small pieces of bone came away, the largest of which was about the size of a split pea. These pieces, insignificant as they seem, if they had not had a free passage kept open for them, could, and in all probability would, have caused a recurrence of those discharging sinuses, so diagnostic of necrosis.

In the case of C. S., (V,) the necrosis was superficial, on the anterior border of the tibia, and at first it was not deemed necessary to use the wax tent, but after three weeks the lint caused so much inflammation around the wound, that it was changed for the wax. At the next clinic a great improvement was visible, the inflammation and tenderness had nearly subsided, and in a month more the last spicula and flake of dead bone had exfoliated, and the patient was discharged cured. This case was remarkable chiefly on account of the large extent of superficial ulceration as compared with the small amount of bone disease. Again, another fact worthy of remark is, that they are so often caused by skating. A young man skates for several hours on a very cold day, comes home chilled through, and goes through the familiar history of necrosis. All of these cases are young men or boys, and three of the four contracted the disease by skating.

VII. A sailor, middle aged, married early in life, and strictly virtuous (he said,) presented himself on May 21st, to be relieved of stricture of the urethra. He had been hit, some eight months previously, by a boom, on the upper and inner part of the thigh and perineum, and very soon after experienced difficulty in passing his urine. It was impossible to pass a moderate-sized sound. The patient was put profoundly under the influence of æther and after much time and manipulation the point of a Holt's dilator was made to engage in the contracted canal of the urethra, after the lacunæ and culs-de-sac had been filled by the ends of fine flexible bougies. It was then pushed into the bladder, and the stricture forcibly dilated. Then a set of Van Buren's conical pointed sounds were passed into the bladder, beginning at four and ending at ten. He was cared for every day till a number sixteen was passed. But nature not having manufactured the orifice of his urethra with due reference to the size of a number sixteen sound, he was put back to a number twelve. This he was taught to use himself, and on June 4th, he introduced it into his bladder before the class, and with perfect impunity to his meatus urinarius. He expressed his delight and surprise at the ease and copiousness of micturition after the dilatation.

VIII. June 11, 1870, a weak, sickly boy, aged 15, residing on Market street, was brought before the class, to be relieved of traumatic stricture. One stricture was found immediately behind the meatus, and was first forcibly dilated by Holt's dilator, and then by the sound. Then a second stricture was found in the membranous portion of the urethra, and was treated in the same way as the first. The boy's development was very small for his age, so that a number four sound was considered as large as it was best to pass in his case. On reaching the bladder the sound gave distinct and audible evidence to the class that the two strictures were further complicated by the existence of a vesical calculus. The lad was sent home to prepare for the operation of lithotomy. On the 18th of June he again presented himself at the clinic. The lateral operation was made, but the stone repeatedly crumbled and slipped off in the attempts to remove it with the forceps. At last the nucleus, with a portion of the surrounding concretion, was withdrawn, and a large quantity of fungus, thickly studded with the same concretion as the main calculus, was scraped from the walls of the bladder. The calculus was also attached to the upper vesical wall. The patient made a good recovery, passing from time to time shreds of fungus similar to those scraped from the bladder during the operation. This patient will, probably, appear again for further treatment of the strictures.

